# Supplementary material for: Barriers to accessing perinatal mental health services in ethnically diverse women in the UK
Source: BMC Psychiatry. 2026 Jan 29;26:190. doi: 10.1186/s12888-025-07159-7 (PMC12922248; doi:10.1186/s12888-025-07159-7)
Supplement: Supplementary file 1 — Supplementary Material 1. [file 12888_2025_7159_MOESM1_ESM.docx]

Tables

Table 1:

Table 1 Participant’s ethnicity information (n=28)

|  |  |  | n (%) |
| --- | --- | --- | --- |
| Ethnicity | | | |
|  | Black/ Black British | Caribbean | 2 (7.1) |
|  |  | African | 5 (17.8%) |
|  | Total Black ethnicities | | 7 (24.8%) |
|  | Asian/ Asian British | British Pakistani | 6 (21.4%) |
|  |  | British Indian | 6 21.4%) |
|  |  | British Bangladesh | 1 (3.6%) |
|  | Total Asian ethnicities | | 13 (46.3%) |
|  | Mixed ethnicities | | 2 (7.1%) |
|  | Other ethnicities (e.g. Filipino, Italian, Polish, Chinese and North American) | | 6 (21.4%) |
| Age (Mean = 32, range 25-44) | | | |
|  | 25-29 |  | 7 (25.0%) |
|  | 30-34 |  | 13 (46.4%) |
|  | 35-39 |  | 6 (21.4%) |
|  | 40-44 |  | 2 (7.1%) |
| Employment status | | | |
|  | Employed (including self-employed) | | 19 (77.9%) |
|  | Unemployed | | 9 (32.1%) |
| Length of stay in the UK. | | | |
|  | UK Born |  | 16 (57.1%) |
|  | 1-5 years |  | 6 (21.4%) |
|  | 6-10 years |  | 3 (10.7%) |
|  | 11-20 years |  | 2 (7.1%) |
|  | 21-30 years |  | 1 (3.5% |
| Number of children | | | |
|  | 1 |  | 14 (50%) |
|  | 2 |  | 11 (39%) |
|  | 3 |  | 1 (3.5%) |
|  | 4 |  | 1 (3.5%) |
|  | Currently pregnant |  | 1 (3.5%) |

Table 2: Main study themes

| Themes | Subthemes | Description |
| --- | --- | --- |
| Cultural beliefs and expectations | Beliefs and perceptions on PMH  Expectations of motherhood  Naming PMH | This theme highlights the diverse perceptions of perinatal mental health among the few ethnicities represented in the study. A common thread across these ethnic groups is the tendency to overlook cases of perinatal mental health, often dismissing them as typical struggles of motherhood. For some women, these challenges are further intensified as they navigate the balance between British culture and their religious beliefs. Consequently, due to this negative perception, there is often a lack of terminology to adequately capture the full range of symptoms associated with perinatal mental health. This absence of language makes it difficult for women to articulate their experiences to healthcare providers. |
| Help-seeking decision | Support structures  Faith as a coping strategy  Stigma and judgement within the community. | This theme highlights the experiences of women who sought support from their families and friends. However, due to a limited understanding of perinatal mental health within the broader community, these women frequently encountered inadequate support. Their struggles were often dismissed as mere stress or even attributed to spiritual issues, which led some to receive advice to pray instead. In the South Asian community, there is a tendency to seek help outside of their familiar circles, driven by concerns about stigma and judgment. |
| Barriers related to accessibility to perinatal mental health services | Women’s experiences with health professionals | This theme highlights a distinction between women who seek help and those who do not. Women who forgo seeking assistance often cite past negative experiences with healthcare professionals, which may include traumatic encounters, discriminatory behaviours, and a general lack of trust. In contrast, those who do attempt to seek help frequently encounter dismissive attitudes, unresolved concerns, and a failure by healthcare professionals to recognize mental health struggles. These negative experiences deter women from pursuing assistance, and for those who do reach out, their symptoms are often minimized or inadequately addressed. |
| Positive experiences with Peer-support groups | NIl | Women reported that they found effective support within the peer support group, as it provided an environment where they could articulate their thoughts and feelings freely. The presence of other women facing similar challenges facilitated open and honest discussions. Moreover, the group's structure and approach were closely aligned with their religious values, which further reinforced a sense of comfort and acceptance. This combination of shared experiences and spiritual alignment allowed participants to engage meaningfully, fostering a strong sense of community and mutual support.. |
