# Supplementary material for: Barriers to accessing perinatal mental health services in ethnically diverse women in the UK
Source: BMC Psychiatry. 2026 Jan 29;26:190. doi: 10.1186/s12888-025-07159-7 (PMC12922248; doi:10.1186/s12888-025-07159-7)
Supplement: Supplementary file 2 — Supplementary Material 2. [file 12888_2025_7159_MOESM2_ESM.docx]

**ESMI-II Topic Guide - Mothers**

*Thank you for taking the time to speak with me. We are interested to know about your mental health journey during pregnancy and in the years after you had your baby. We know that each person’s journey is unique, so we are interested to know about you as a person, and any positive or negative experiences you’ve had that may have influenced your journey. And importantly, we’d like to hear about the experience of any care you may have received from health professionals or other organisations, including what has worked or not worked for you, and how you think the care has impacted on you, your family and those who you are close to.*

*We are conducting these interviews with a range of mothers across the country to help identify the best ways to support families during this time. There are no right or wrong answers so please be as open as you feel comfortable with. We are not linked with the services in any way so anything you do say to me will not affect the care you receive. Everything you say to me is confidential and when we write up the results and recommendations no identifiable information will be used. The only time we would have to pass on information would be if we had major concerns about your safety or the safety of others.*

*We understand that these questions are personal in nature, and sometimes may be difficult to talk about. Please know that if at any point you do not want to answer any part or any question, you do not have to. We can also take a break at any point as well if you would like to.*

*First, is there someone you would like to nominate for me to contact if you do get upset. The interview should last about an hour and a half and I would like to record it with your permission. Do you have any questions before we begin?*

**Ensure participant has read the information sheet and ask them to sign the consent form if they are happy to proceed.**

**Start recording**

*NB. These topics and prompts may be modified following interviews with community leads.*

*Things we want to explore: sociodemographic, home situation, marital status, financial, presence of other conditions.*

***Topic 1: Background and perception of mental health***

Tell me a little about you, your background, what is your living situation, support network, etc? (home, living situation, finances, access to resources, etc).

What cultural views of motherhood are held in their community?

How do you seek support?

Do you think there are any barriers to seeking support related to cultural factors?

What kind of mother did you want to be?

Did you have the right support to achieve this from your community/family?

How did you feel about being a mother?

Did you experience any difficulties? Tell me some of the challenges

What is your perception of mental health? How is this viewed in your culture?

Do you have any special name given to these symptoms.

When is it appropriate to get help?

Where would you seek help? Who would you trust?

**Topic 2: Journey through the perinatal period**

How were you feeling during your pregnancy?

PROBES: Was there anything going on that impacted on how you were feeling?

Did anyone around you notice any changes in you?

Did you speak to anyone about how you were feeling?

Who did you tell?

And how did they respond?

Did you think you needed help or did anyone around you think you needed help?

Did you feel supported at home? What your home life like?

During your midwife appointments were you asked about how you were feeling?

If yes, how did they ask about it? Did you feel comfortable responding to it?

If no, would you have wanted them to ask you about it? How would you have wanted them to ask you (word it appropriately)?

Did you mention how you were feeling to your health visitor/ midwife?

PROBES: What did they ask you during appointments? Did you feel like your midwife listened to what you needed? Did they act on what you say you needed/ was telling them? Did you feel it was the right response?

Was there anything in the labour that stood out, that did or didn’t go well? Or was particularly difficult? Or that was positive?

Who was there during the labour and how were they treated?

Did you have the people you wanted around you during the birth? Who was there?

Did you feel equipped with informations you needed? Did you have the people around you who could find the information you needed?

How did care you received now compare to previous pregnancies?

**Topic 2: Services accessed for support**

What services were accessed this time?

(If answered no) How did you cope during this time?

(If answered yes) How did you get to know about the service (Referral)

Did you experience any barriers (e.g. cultural, organisational) to accessing services?

What additional help would have been useful? (if they mention mental health problems probe about accessing help for this)

How was the service helped? What did they provide?

If they received Perinatal mental health support:

How did you hear about perinatal mental health services

Were you referred or you willingly sought help for your self

Did you think the services were supportive.

What was helpful and unhelpful about these services?

Did you think your expectations were met?

If not, what were your expectations.

How can care be more tailored to your need: culture, religious and social

**Is there anything else you would like to tell us about that we haven’t asked you?**

**[END RECORDING]**
