# Supplementary material for: Barriers to accessing perinatal mental health services in ethnically diverse women in the UK
Source: BMC Psychiatry. 2026 Jan 29;26:190. doi: 10.1186/s12888-025-07159-7 (PMC12922248; doi:10.1186/s12888-025-07159-7)
Supplement: Supplementary file 3 — Supplementary Material 3. [file 12888_2025_7159_MOESM3_ESM.docx]

**Barriers to Accessing Perinatal Mental Health Services in Ethnic Minority Women in the UK**

Ebunoluwa Makinde*^1^, Antoinette Davey^1^,Gina Collins^1^, Geoff Wong^2^, Sarah Morgan-Trimmer^3^, Chris McCree^4^, Jo Brook^5^, Louise Fisher^6^, Helen Sharp^6^, Louise M Howard^7^, Heather O’Mahen^1^

*Corresponding author: Ebunoluwa Makinde, University of Exeter, Washington Singer Laboratories, Perry Road, Exeter, EX4 4QG. E.T.Makinde@exeter.ac.uk

1. Mood Disorders Centre. Department of Psychology, University of Exeter, Perry Road, Prince of Wales Road, Exeter, EX4 4QG, UK

2. Nuffield Department of Primary Care Health Sciences, University of Oxford, Oxford, UK

3. Department of Health and Community Sciences, Faculty of Health and Life Sciences, University of Exeter, Exeter, UK.

4. Centre for Parent and Child Support and Community Perinatal Services, South London and Maudsley NHS Trust, London, UK

5. The Tavistock and Portman NHS Foundation Trust, London, UK

6. Department of Primary Care and Mental Health, Institute of Population Health, Faculty of Health and Life Sciences, University of Liverpool, Liverpool, UK.

7.Section of Women’s Mental Health, Institute of Psychiatry, Psychology & Neuroscience, King’s College London, UK

**Abstract**

**Background**

Mental health problems are common among women during the perinatal period (here defined as during pregnancy and the 2 years after birth), affecting up to 20% of women. Although there are effective treatments for perinatal mental health problems, ethnic minority women who are at higher risk of developing perinatal mental health problems are less likely to access treatment for their perinatal mental health problems. This study explored perceptions of perinatal mental health problems and barriers to accessing treatment amongst a sample of ethnic minority women with current or past experiences who did not receive help from a specialist team.

**Methods**

This study is qualitative primary research. Twenty-eight women living in England who reported they struggled with emotional and mental health challenges during their perinatal period but did not receive assistance from the specialist perinatal mental health team were recruited from community groups and adverts. Women participated in online interviews, which were transcribed. NVIVO was used to support a thematic analysis.

**Results**

The study revealed that substantial barriers to seeking help arise from women's interactions with their healthcare professionals. When women feel a lack of trust, experience discrimination, or sense judgment from healthcare providers, they are less inclined to pursue assistance. However, the motivation to seek help is significantly shaped by community perceptions of perinatal mental health issues, particularly for those women who have made efforts to access support. Women had a positive experience in peer support groups

**Conclusion**

A significant barrier to accessing perinatal mental health services for ethnic minority women is experiences with health professionals. Moreover, women from ethnic minority groups may encounter additional problems regarding help-seeking due to cultural influences and attitudes towards mental health and motherhood roles. Hence, the intricate interplay between cultural factors and healthcare experiences highlights the urgent need for a more culturally competent approach to perinatal mental health services.

Key words. ethnic minorities, mental health, maternal health, access to health care

**Introduction**

Perinatal mental health problems affect between 10-20% of women, and rates may be higher in women from ethnic minority groups (1,2). The impact of these perinatal mental health problems is significant. They are associated with obstetric complications, poor neonatal outcomes (3), maternal disability (4), suicide (5,6) and postnatally increased risk of negative infant social, emotional and cognitive outcomes (5). There are effective treatments for perinatal mental health problems (7), but there are inequities in access to these treatments. Women from ethnic minority groups are also less likely to report their symptoms to health professionals (2) and when they do, less likely to receive referrals and/or access support (9).

Understanding ethnic minority women’s journey to perinatal mental health care treatment involves having knowledge of the unique factors that contribute to the development of their mental health problems, as well as the influence of cultural perspectives of mental health issues. These factors include discrimination, immigration problems, economic instability (11) and inequities in problem recognition and referral by health professionals (9). Thus, most ethnic minority women who have mental health problems may feel wary and untrusting of the health system because of their previous poor experiences of these systems (12). In the perinatal period, ethnic minority women’s cautious viewpoints of healthcare providers may crystallise, as health conditions and unfavourable encounters with health services combine to exacerbate negative health outcomes (12). These encounters may include discrimination and dismissive attitudes, which are most likely to happen in the UK to women from Black and South Asian backgrounds (12). Their negative health outcomes, and the lack of support they may have received leave ethnic minority women at risk of worsening mental health and traumatic experiences, yet their prior interactions with health professionals are a key factor determining women’s willingness to access future healthcare services (10).

Ethnic minority women’s pathways into mental health treatment are further compounded by obstacles that exist across different levels, with barriers at individual, sociocultural, organizational, or structural levels (13) and may include cultural differences between healthcare providers and women seeking assistance, lack of culturally sensitive care and differences in their understanding of perinatal mental health compared to that of their healthcare providers. Critically it is also important to consider how cultural factors can interact with other disadvantages, including having lower income and facing multiple physical and mental health morbidities (12).

Recently in England, the NHS invested over £365 million to expand perinatal community mental health teams with the aim to provide equitable access to treatment across geographic regions (14). Despite these investments, inequities in access for women from diverse background remain. It is therefore important to understand the factors affecting the underlying ongoing inequitable access to support. Recent qualitative studies have highlighted barriers to care amongst ethnic minority women who accessed perinatal mental health treatment (9,10) but there is a lack of studies examining the experiences of ethnic minority women who have experienced mental health problems during the perinatal period but did not access care. It is important to understand if this group of women face unique barriers to perinatal mental health care to improve equitable access to care.

**Methods**

*Aim*

The study aimed to qualitatively explore perceptions of perinatal mental health problems from ethnic minority women who did not access specialist care and their perceived barriers to accessing specialist perinatal mental health care.

*Participants and Recruitment*

The sampling method for this study lies within the purposive sampling technique. Although the study was advertised and potential participants were asked to reach out, participants were selected based on the pre-defined inclusion criteria for credibility and richness (Gentles, et al., 2015). Participants (n=28) were recruited via study fliers that were advertised through social media platforms, targeting parenting groups. Fliers also were sent to voluntary and community sector agencies (VCS) to facilitate the recruitment of women within their support networks. ~~Author EM, who is from Nigeria and a first-generation immigrant, a mother and a doctoral candidate with interests in perinatal health and child health, attended events and sessions run by VCS groups where the study was publicized~~. Participants who responded to the invitation were screened for eligibility. Inclusion criteria included: gave birth within the past four years, residing in England, experienced self-defined mental health problems during pregnancy or the 2 years following childbirth (as NHS England provides perinatal specific care within this period, (14)), not received formal mental health support during this period (though they may have attempted to seek help). The researcher (EM) verbally reviewed consent and provided eligible participants with a link to the participant information sheet and consent. All participants provided electronic consent before the interview commenced. Interviews were conducted online using video conferencing (Zoom). Participant’s ethnicity information is described in Table 1.

*Procedures*

A semi-structured interview guide was developed by EM, AD and HOM, with input from the other listed authors of the study and the study’s Participant Advisory Group (PAG). The PAG consisted of women who had experienced perinatal mental health (PMH) problems and included women from ethnic minority groups, including South Asian and Black women (see Supplementary material 2 for the topic guide). The interview guide included questions about women’s views of their community’s perception of PMH and motherhood, perceived barriers to seeking PMH support, their own PMH experiences, including their experiences with health professionals, and ways they thought services could improve to meet their needs. All participants were provided with an information sheet outlining the purpose of the study, any potential risks and benefits of taking part, and how their data was to be collected and used. Prior to the interview participants were informed of their right to not answer any question they did not want to, and if they wanted to discontinue at any point they were able to do so. If participants became upset at any point during or after their interview the study researcher could offer them support, direct them to appropriate services and if needed trigger the study safety protocol, which included contacting the participants’ GP if necessary. Participants who sought help from NHS mental health services were asked about their experiences and if they received any help. EM conducted all interviews (lasting between 35-73 minutes) between October and December 2022. Forty percent of the participants had their children present during the interview, but care was taken at the beginning of each interview to ensure the interviewee had privacy. There were no apparent signs of the presence of any other adults in any of the interview sessions. Six of the participants were still on maternity leave at the time of the interview, and one of the participants was pregnant during the interview. Participants were renumerated £25 for their time.

*Data Analysis*

All interviews (over Zoom) were audio-recorded by the study researcher and transcribed verbatim by an external transcriptionist who had signed a confidentiality agreement with the research team Thematic analysis techniques were used to identify key themes and patterns from the interview transcripts through reading and re-reading (Rice & Ezzy, 199). The thematic analysis technique gives room for inductive and deductive analysis approaches employed in this study. The deductive approach initially drew upon existing research for preliminary ideas and organisation, and the inductive approach gave room for participants' voices, which were iteratively identified through an assignment of codes. At the initial stage, transcripts were read and re-read for data familiarisation, and a coding framework was developed from the first transcript. Subsequently, the same coding scheme was consistently applied throughout the data, with additional codes being developed as new concepts emerged. To ensure reliability, to ensure reliability, AD also read transcripts and coded interview transcripts. The two coders identified patterns in the codes, which were then categorised under sub-headings and developed into themes. The two coders met with the remaining analysis team ( HOM & GC) to discuss the codebook and reach a consensus on the emerging themes (Braun & Clarke, 2006). In addition, the interviews were analysed on a continuous basis and once the team felt that no new themes were present and the data collected captured the diversity, depth and nuances of the concepts we were studying saturation was established. ~~Thematic analysis was used to analyse the data. Using an iterative approach of inductive and deductive thematic analysis, as outlined by~~ ~~Braun and Clarke (2006)(15), the lead researcher, EM, immersed herself in the data through in-depth reading of the transcripts. She then generated codes, which were discussed iteratively with HOM, GC and AD until a consensus of the coding system was reached.~~

An initial thematic model comprising themes and subthemes was developed in consultation with EM, HOM, GC, AD, GW, SMT, HS, LF, CM, and JB. As new questions emerged from these nascent themes, the interview guide was iteratively modified. In addition, themes were considered in the context of existing perinatal mental health literature, professional clinical expertise, and a stakeholder event in London (attendees included academics, NHS clinicians, representatives from NIHR, NHS England, individuals from the voluntary and community sector) where the initial model was presented and stakeholder and lived experiences (our PAG group) views were sought. The stakeholder event presented the initial findings providing the audience an opportunity to feedback and comment. The final themes were then established considering these sources. A valuable contribution from the stakeholder's event was the significant role that peer support groups play in supporting women during the struggle time.

*Reflexivity*

Ten authors of the study self-identify as female (EM, HOM, AD, GC, JB, HS, LF, CM, SM, LH), and one author identified as male (GW). Of all study authors, AD, GW & EM are from ethnic minority backgrounds, and EM, HOM, and GW are first-generation immigrants, and LH and SMT are second-generation immigrant. The core analysis team self-identify as female (EM, HOM, AD, GC) and are all mothers. Three of the authors come from a psychology background and are also therapists and EM is from a public health background. EM, being aware of her ethnicity, experiences, and background, checked her perspectives against other members of the team, some of whom were immigrants and others who were not. Thus, the analysis considered the team’s background when reviewing participant’s accounts of their experiences. Several meetings were held to review the results and analysis process to ensure the validity of the analysis. Given our differing backgrounds in the research team, we all agreed with Lincoln and Guba (1985) that reality is socially, culturally, and historically constructed. We acknowledged that our backgrounds, values, and beliefs may influence data collection and interpretation. Hence, we played our role as researchers and allowed the data to emerge through interviews with our participants. Moreso, as no reality exists in its form, but through human construction and interpretation ( Smith, 1983), hence we take steps to minimise this by keeping memos and meeting continuously with the wider research team

**Results**

*Participant characteristics*

Participant demographics are described in Table 1. Slightly fewer than half of the participants were from an Asian background, a quarter were African/Afro-Caribbean and almost a third were from mixed or “other” heritage (e.g., Chinese, Italian, Filipino, North American and Polish). Approximately half of the participants were first-generation immigrants, with 20% having been in the UK for less than five years. Half of the participants had one child, while just under half had two children, and one participant was pregnant at the time of the interview. We considered births that occurred within the last four years (2018-2022); however, if there were two births during this period, we focused on the most recent one. None of the study participants required an interpreting service as they had conversational English. Less than half of the participants attempted to seek help for their mental health difficulties, though they ultimately did not access formal mental health care. Pseudonyms were used in place of participant’s real names.

<<INSERT TABLE 1>>

*Overview of themes*

The motivations and decisions to seek help for perinatal mental health disorders for ethnic minority women in this study were influenced by their ethnic backgrounds. Cultural beliefs, practices, and values significantly shape their perspectives on mental health and the stigma attached to it. Additionally, the interactions these women have with healthcare professionals throughout their journey present significant obstacles to seeking the help they require. Negative experiences, such as a lack of understanding or sensitivity from providers, discrimination, and lack of trust discouraged women from reaching out for support, thereby complicating their paths to recovery. Four themes emerged from this study, namely: Cultural beliefs and expectations, Help-seeking patterns, accessibility barriers and positive experiences with peer support (see Figure 1). Subthemes further characterised three themes focusing on barriers; Within the cultural beliefs and expectations theme, there were three subthemes. The first subtheme was beliefs and perceptions of PMH, where women described their ethnic group’s view of mental health. The second sub-theme was the expectation of motherhood, characterised by women’s descriptions of their expectations of motherhood within their culture, and how these conceptions could prevent families from recognising symptoms of perinatal mental health issues. The final subtheme within this theme was the inability of participants to name perinatal mental health problems in their language thus making it difficult to ascribe meanings to their symptoms and communicate their symptoms to health professionals.

The second theme of the study is the help-seeking decision, characterised by the following sub-themes: support structure, faith as a coping strategy, and stigma and judgment within the community. The final theme was barriers related to accessibility, which was captured under the subtheme: women’s experiences with health professionals (e.g., midwives, general practitioners, health visitors).

Because women from ethnic minority groups have emphasized the uniqueness of their different cultural backgrounds and communities in their experiences, we report the results by different groupings: Black/African; South Asian, and “other.” We recognise considerable variability within these communities but hope to begin to detail some of the nuances of difference within these cultures while also highlighting commonalities with this approach. Given the study’s sample size within the ethnic category, drawing broad conclusions may be challenging. However, the robust method used in the study yielded consistent results with existing findings hence the findings from this study can be applied to similar settings

<<INSERT FIGURE 1>>

**Cultural beliefs and expectations**

*Beliefs and perceptions on perinatal mental health*

Most women in the study demonstrated an awareness of perinatal mental health issues. Some had acquired awareness of perinatal mental health through work experience, social media platforms, and past mental health experiences. Despite this awareness, participants discussed how perinatal mental health problems within their communities remain a “taboo” topic, one that is largely unrecognised by older generations in their communities. Views from different ethnicities are provided below.

Women from Black ethnic groups reported that mental health problems were largely unrecognised in their community and more emphasis was placed on physical wellbeing by the community.

*“I think in my culture it’s not everybody that understands that there is something called mental health, it's possible for you to look well on the outside but on the inside, you don't look well.” Ade (African)*

Women from South Asian communities expressed a similar view to the women of Black ethnicity, however, there was a subtle difference in that mental health was seen as stigmatizing in their community. They described how mental health is not spoken of and is yet to be recognised, hence little importance is placed on mental health wellbeing.

*“I come from an Indian community, it’s not very good. [laughs] Mental health, they think it’s not very important, and it's almost frowned upon. It's like, "Oh, well, what's wrong with her?" It's about tough love. You have to be strong all the time. You can't cry, and you can't have a bad day. You're a mom now, you have to get on with it.” Limra (Asian)*

The quotes from women from the ‘other ethnicities’ depict some awareness of mental health; however, it is mostly ignored as it is taboo subject or people interpret it as weakness and would not wish to draw attention to it.

*“In Latin America, mental health is not something you discuss. It's something that is taboo.” Justina (South American)*

*“In Italy, if you talk about Mental health it is perceived as weakness; people wouldn’t really want to talk about their own weakness.” Gioia (European)*

Little variation was noted in perceptions of mental health between ethnicities and across each group; symptoms were mostly ignored and not prioritised as much as physical health needs. In some cases, mental health symptoms were normalised and seen as normal phases in life. It is also re-interpreted by others in their community as being due primarily to physical causes. Women reported these beliefs were passed down from older generations, thus impacting the confidence of most women to seek help for their mental health needs. While they were aware of the symptoms, they were torn between interpreting them as normal life processes, which affected their decision-making and timing in seeking help.

*Expectations of motherhood*

Women discussed their perceptions of expectations placed on motherhood by broader society. A key theme that emerged was how women struggled with compound expectations: balancing British mainstream motherhood expectations with the motherhood expectations of their community. These expectations were further complicated by their community’s beliefs and perceptions of perinatal mental health problems.

Women from Black ethnicity discussed community beliefs about being a “strong Black woman,” and how these built-up expectations that one could not admit to any “weakness” (i.e., mental health problems).

*“You are a strong Black woman, which will mean you are going to be a strong Black mom. I feel like that coat is a very dangerous one to wear because you are allowed to be vulnerable and because you’ve been given that coat, that very strong responsibility, you feel like you can’t let that down and that can contribute very much back to Mental health.” Bernice (Black)*

Women from South Asian backgrounds expressed a sense of responsibility in training their children according to cultural norms. The pressure was especially felt by women who strove to instil religious beliefs in their children. They found it challenging to meet this expectation due to the perception of the broader UK environment as non-religious or conflicting with their religious values.

*“Being a Muslim mother, I need to teach every single thing like our values what we need to do. I am doing the best what I can do. Look we are in western country, so we are in between at the moment. We need to teach them our values and culture as well.” Harini (Asian)*

Within the Italian community, the role of a mother was compared to that of the Virgin Mary, known for her sacrificial nature. As a result, motherhood was seen as a responsibility centred around selflessness and setting a positive example for one's children.

*“The mother is very much a Christian model of motherhood, Virgin Mary, all sacrificial. You give it all for your kids and for your family. Also, that means that if you feel low or anything, it's something that you have to work on yourself because you have to give your everything to your kids. You have to give everything to your family.” Gioia (European)*

In this study, all the women expressed their identification with the challenges of motherhood and the aspiration to follow examples set by others. They aimed to avoid being seen as inadequate in their role or criticized as ungrateful mothers when they encountered obstacles, and therefore prioritised efforts to meet these expectations. This struggle was especially daunting for women who desired to educate their children in religious customs because they perceived strong conflicts between aspects of UK culture and their religious customs. Additionally, they feared failing not only in their maternal responsibilities but also in their social environment. In some cases, these conflicts and the burden of their responsibilities could contribute to problems with their mood, but admitting to their emotional health struggles could serve to confirm their failures.

*Naming perinatal mental health*

Across different ethnic communities, participants reported not having a term or name for perinatal mental health in their language and as a result women often lacked the terminology to describe their symptoms to families and their health professionals. The 50% of women in this study who were first-generation immigrants described how challenging it was to use English, which was often not their first language, with health care providers, coupled with the fact that in their cultures they frequently lacked clear names for perinatal mental health problems. This added to their suffering because they did not have a way to label and understand what was happening to them (as seen in Black ethnic groups where symptoms were perceived as stress and in Italian communities, symptoms were perceived as weaknesses).

*“Even though I noticed that something was wrong, it was even difficult for me to articulate what was wrong.” Ade (Black)*

*“Mental health is worse, if you feel pain in your body you can tell. Like I got a pain in my leg or arm, but mental health is worse.” Harini ( Asian)*

In other circumstances, the lack of cultural terms to accurately describe their experiences impacted their ability to discuss their experiences with healthcare providers, sometimes leading to misunderstandings with others that felt threatening to the women.

*“I remember one day saying to the health visitor that-- like saying to her that--she [the baby] was crying and I just found myself like I couldn't cope almost. I just got frustrated and she [the health visitor] immediately took it the other way. Like, "Oh, what did you do when you got frustrated? I said, "No, I didn't get frustrated with her. I got frustrated with the situation because it's 4:00 AM and I'm tired, my body's aching and I'm trying to feed her. She's not going to sleep.” Gioia (European)*

In this instance, the woman felt that the health visitor was beginning to worry that she might respond inappropriately to her infant when tired and overwhelmed, whereas the woman was attempting to describe how exhausting and challenging crying could be, especially late at night.

**Help-seeking decision**

Women discussed how their perceptions of mental health and expectations of motherhood impacted their decisions to seek formal perinatal mental health support. Variations in ethnic help-seeking patterns were identified as a key barrier in this process. From women’s accounts, women from the South Asian community were more likely to seek help outside their support networks and families for fear of discrimination and judgment within their community. In contrast, most women from other ethnicities described seeking support from within their families and support networks because they felt that symptoms of mental health were either initially misinterpreted by others (e.g., health professionals) or because they had strong family orientations and despite fearing family might struggle to understand, saw them as their first and safest place to find support. Views across different ethnicities are presented below.

*Support structures*

Women reported that they faced culturally reinforced responsibilities to be “strong” mothers and mental health problems were cast as “stress from adjusting to motherhood” or a ‘spiritual attack’ (demon possessed). They felt deterred from seeking formal help for their mental health needs and instead focused primarily on seeking support within their own support networks (church members or friends) and with their families.

*“When I asked my mom, she just believed it was just because of the pressure of the baby, so the baby is crying, and you can’t sleep at night. My mom just feels like it is just stress. She believes that it is more of physical stress, much more than mental stress.” Pearl (Black).*

*“In our culture, taking care of your emotions would really boil down to that person. Because we have very good family support, everyone is there, all the grandparents and the extended family, you would have someone to talk to. Speaking with therapists or seeking mental health support or emotional health support is not as frequent, it’s more of the immediate family circle, whomever you are closest with, then that is where you get your support from.” Suzanna (Other ethnicity- Filipino).*

In our study, we found that most ethnic groups represented, except women from South Asian backgrounds, predominantly sought support from their close networks and family members during challenging times. They described their communities as largely family-oriented, therefore they stated they would typically seek initial support from the people who made up their support system. However, women said they often failed to find adequate support from within their families and friend networks because of limited knowledge of perinatal mental health existing within the community.

*Faith as a coping strategy*

in instances where the necessary support was lacking or insufficient, religious-oriented women used their faith or were encouraged by family members and friends to turn to their faith and engage in prayer to cope with their difficulties.

*“I’m a Christian, so I take everything to God in prayers. [laughs] If I'm feeling down, sometimes I talk to God exactly about how I'm feeling, and it helps me get better.” Ade (Black)*

*“I would pray because I'm Muslim, I would pray, I would-- Thankfully, I had my mom, but moms have other commitments too.*” Limra (Asian).

This reliance on spiritual practices highlights the significance of faith as a source of strength and resilience in the absence of adequate support from close networks

*Stigma and judgement within communities*

Women from Asian communities were more likely to describe facing stigma and judgment from their family and community for having mental health problems. As a result, they reported that they were more willing to consider external support for their mental health if it was outside their support networks and relations. These women tended to use services from peer support groups more than those from other ethnicities.

*“I think the safer option for a lot of people is just to tap into somebody that's not someone they know because with mental health and wellbeing, sometimes it's a very sensitive topic and people can misinterpret it and it's just not something that's always understood. I feel like then that's why people tap into maybe mainly other sources.” Kaiya (Asian)*

*“If you go to your own community or mosque, they judge you. They ask you so many questions.” Harini (Asian)*

Many South Asian women were afraid to share their experiences with their family members because they feared being judged as a bad mother and facing discrimination and stigma. These moms also believed that seeking help within their community might not yield positive results as most people do not know about perinatal mental health and how to provide support during this period. As a result, seeking assistance from parent groups or children play centres where they could find other moms and peer support groups (both physical and online) was a common trend among the South Asian community or instead they would just keep it to themselves. However, one participant expressed a desire to seek help from her family but could not be due to other issues going on within her family.

*“I don't feel like I could speak to my family because they're grieving themselves and I just don't like to burden my own family with my issues. I've never talked to them about them that much.” Kaiya (Asian)*

**Barriers related to accessibility to perinatal mental health services**

*Women’s perceptions of health professionals’ attitudes*

Although cultural perceptions of mental health and help-seeking were significant barriers to accessing formal perinatal mental health services, women’s willingness to seek help was also significantly impacted by their past negative experiences with health professionals. Notable amongst the narratives of women in this study were both personal and community experiences of discrimination from health professionals that resulted in cautious and at times fearful perceptions of formal mental health services. Most women in this study described their experiences with their health professionals as emotionally difficult because of unmet expectations and needs. This was especially notable within the maternity setting, with some women describing traumatic and medically neglectful birth experiences resulting from interactions with maternity health professionals. In two instances, women felt they were refused referral for mental health treatment as they felt the staff’s medical errors may be exposed. These experiences were not specific to a particular ethnic group.

*“I felt like even when I made complaint to that matron who came to see me, I felt like she even judged me. There was nothing like we will investigate into this why this happened to you. There was nothing like we should do an incident form about it. There was nobody else to go to, because these are the actual professionals who can refer you to other places. If these professionals behave, have this attitude, where can a woman go” Isla (Asian)*

*~~IR008027~~*

*I just felt I'd got to a stage where I didn't know who to keep reaching out to because I just felt I was a broken record. I kept telling everybody about how I was feeling. Everyone kept saying, wow, this is a really difficult thing that you've been through, but no one was actually helping to do anything I think I just gave up. ”Kana (Asian)*

Most women in the study felt that their health professionals did not meet their expectations during their maternity experience. They believed that midwives and nurses in maternity units should be trained to identify mental health issues as many were unable to detect signs of mental health problems or ignored them when symptoms were present.

*“I spoke to a lot of healthcare professionals while I was in hospital. Some I was very obvious and open with how I was feeling, and some could have asked a few more questions for me to open up. They've [health professionals] done nothing about it. If they have had the training, and I think that's the first bit is you need to have the staff or a specific person that goes around to every mother in that ward. "How are you feeling today? How is your mental health." Kana (S. Asian)*

A significant number of women shared their apprehensions about seeking medical advice due to their lack of trust in healthcare professionals. They were also afraid of being reported to social services if they disclose their symptoms.

*“I had this idea that if the midwife doesn't like something that they hear, they're going to take your children away from you.” Keon (Asian)*

If women did overcome these fears and approached health professionals for their mental health needs, they reported they felt as if the health professionals were dismissive towards their needs because of their ethnicity. This was especially true for Black women. They felt as if their fears of discrimination were realised.

*“I feel like it would have been different if someone from another background had come to them and say it the way it is because I couldn’t call it the way it should be called. Maybe that was why I couldn’t get the help that I needed.” Chelsea (Black)*

*“I feel like with Black people especially, that happens, they feel like you should be strong, and you should be to get through it. They don't really offer you-- I don't know. The way I see it, if I were White, they probably would've offered me more help.” Charlene (Black)*

Some women who described having mental health problems because of the trauma they experienced caused by health professionals were reluctant to pursue formal mental health support because they perceived mental health professionals as being representative of the types of individuals who had traumatized them in the first instance.

*“I was saying, they were saying that my wound is fine, but in the end, it was septic all along. Now after that I just don't trust, if I get unwell, I wouldn't think to go and get medical help. I'd try and sort it out myself.” Isabella (Black)*

**Positive experience with peer support groups**

Out of the twenty-eight women who were interviewed, a little less than half of the participants sought assistance from universal mental health services but were unable to receive help from formal perinatal mental health services due to reasons such as waiting times or dismissive attitudes from health professionals. Of the total participants, thirteen utilised support provided by their local VCS support groups or online support groups, without the help of mental health services. Women described the support groups as their happy, safe havens where different forms of support were available and where their fears of motherhood were voiced and validated. Women also described their different support groups as free of judgment and discrimination, places where their cultural values and beliefs were respected.

*I didn't want to say certain things because the GP or that health professional would look at me like, I'm a crazy Black woman and I don't know how to handle my emotions. She wouldn't get that, but at the minute I said that to someone else that looked like me, they got it instantly.” Bernice (Black)*

*“Then she (peer support coordinator) just told me some things and they were religious, they're linked with my religion in terms of like, they make sense to me because I'm quite strongly in my beliefs in terms of religiously, and then they meant something to me.” Kaiya (Asian)*

According to women's reports, support groups led by individuals who had previously experienced perinatal mental health challenges were particularly effective in helping women during these difficult times. Participants felt represented in these groups because the coordinators shared either their ethnicity or religion, providing them with culturally sensitive support. Additionally, women felt comfortable discussing their fears openly, knowing that other mothers in the group were likely to be going through similar experiences*.*

**Discussion**

This study explored the barriers to accessing perinatal mental health services for ethnic minority women who had not accessed specialist mental health treatment. Three themes focused on the barriers to accessing perinatal mental health services and one theme echoed the positive experiences from peer support groups. The four main themes are defined as follows: cultural beliefs and expectations, help-seeking patterns, barriers to accessibility and positive experiences with peer support groups

*Cultural Beliefs and Perceptions of perinatal mental health*

We developed three subthemes that were relevant to the overarching theme which included beliefs and perceptions about perinatal mental health (PMH), motherhood expectations, and PMH naming. Although there has been a significant increase in PMH knowledge amongst women particularly in regards to identifying PMH symptoms, the current study revealed that more than knowledge of symptoms was needed to encourage help-seeking amongst women from ethnically diverse backgrounds. Ethnic beliefs and perceptions passed down from generations, posed significant barriers to seeking help amongst perinatal women. These findings are consistent with Smith et al. (13), which pointed to the lack of open discussions around PMH amidst women and families leading to help-seeking barriers.

A significant number of participants from this study described an unwillingness to seek formal mental health support because of the fear of being judged as incapable of undertaking their role as a mother by both their families and health professionals. Women from across all ethnic groups expressed worries about judgement and lack of understanding from health professionals, and women from South Asian communities were especially concerned about disclosing to their families as they feared their situations would be dismissed and they would be labelled as incompetent mothers. Previous reviews by Watson, et al., (2019) (5) have described how mental health problems can threaten women’s identities, and, consistent with other research, women described how their mental health problems were often attributed in their communities to physical or situational causes, such as hormonal changes or the care-taking pressures (16–18). Another significant finding from the current research was the interpretation by most women that mental and emotional struggles were considered normal within their communities because they were the consequence of the need to fulfil expected cultural, societal, and religious roles. These beliefs come with different expectations that center around women trying to avoid been labelled as ungrateful or a bad mother by their communities.

*Help-Seeking Patterns*

Although some patterns of fears of discrimination and beliefs about mental health were consistent cross different ethnic groups, we found some variations around help-seeking behaviours between different groups. Black women, for example, were more likely to seek help within their immediate community before accessing formal services. They described strong concerns about discrimination from health professionals resulting in a misinterpretation of their mental health symptoms that could result in a failure to act on their reports and at worst, negative outcomes such as having their child removed. Though these fears are also present in White women (13,19), Black women’s fears were compounded and magnified because of their previous experiences of discrimination. In turning to family first, however, women described how their symptoms were often interpreted as stress, mirroring findings that have also been reported by Edge (2011)(20). As a result, women described waiting to seek formal support, a pattern that is consistent with other studies that have found individuals from Black communities are more likely to delay reporting symptoms to health professionals until symptoms have become severe (17,21,22).

In contrast, South Asian women described worries that their symptoms would not only be misunderstood or misinterpreted by their families and communities, but that they would be strongly negatively judged as a result. Further, some South Asian women reported worries that there was little confidentiality in their communities, so if they told their families, “Everyone would know” and they would face stigma from their entire community. Consequently, they mostly expressed their willingness to seek help from outside their support networks. These finding echoes that of Witkowski et al., (2011) (23) who found that women from Asian backgrounds felt doubly disadvantaged for lack of support within their communities and an inability to get needed help.

*Accessibility Barriers*

The NHS is making an effort to enhance access to perinatal mental health services, aiming for 66,000 women to be able to access perinatal mental health services within 24 months post-conception by the end of 2023/2024 (NHS, 2016). Despite this initiative, our study identified that many women face significant barriers to seeking help often stemming from traumatic experiences with health professionals, these experiences were often characterised by unmet expectations, feelings of discrimination, neglect and lack of trustworthiness. These findings resonate with previous research which noted health professionals are often perceived as unsympathetic (Edge, 2011). Additionally, another study revealed that significant ethnic minorities were not being asked about their mental health Harrison (Harrison, et al., 2023)while some women expressed fears of being stigmatised by their health providers (Smith, et al., 2019). This fragmented relationship and negative experiences often lead to reluctance and fear by women about disclosing their mental health struggles and symptoms because they worry that their symptoms and problems would be misinterpreted by health professionals (5,17). The findings of this study suggest that training opportunities for healthcare professionals regarding why ethnic minority women struggle to seek perinatal mental health support is important as also suggested by other researchers (24,25).

*Positive experiences with peer support groups*

Furthermore, the study findings demonstrate that many women found peer support groups beneficial. Peer support groups provided a secure space where women could openly discuss their concerns, without fear of judgement, and the ability to find people who spoke about emotional problems with a similar language and provide reassurance on issues around PMH. The importance of peer and professional support in a community setting has also been reported by others (5,26). Women concluded that peer support groups are important to them during this study for reasons of safety, validation, and peer learning.

*Clinical Implications*

The implications of our study are noteworthy in highlighting the need for strategies aimed at promoting help-seeking behaviours, identifying training needs of healthcare professionals and improving perinatal mental health outcomes for ethnic minority groups. Healthcare providers and policymakers must acknowledge the influence of cultural beliefs and perceptions, as well as healthcare professionals’ attitudes towards women when designing effective strategies. Addressing these barriers necessitates fostering open discussions surrounding PMH, challenging stigmatization associated with seeking help, and establishing culturally sensitive support systems. By considering the cultural contexts in which individuals exist, healthcare providers can create an environment that encourages women from diverse ethnic backgrounds to seek assistance, address their needs when asked for help and refer them to specialist perinatal mental health services according to their needs.

**Study strengths and limitations.**

The focus of this study addresses an under-researched area, contributing valuable insights into the experiences of ethnic minority populations. The findings highlight significant barriers to seeking help for perinatal mental health issues, which will be beneficial for policymaking, intervention provision, and clinicians' understanding of ethnic minority women. It also underscores the influence of culture on mental health challenges and motivations to seek help

However, the study has limitations, including a narrow recruitment window and a sample size that may not fully represent the diversity within various ethnic groups. Nonetheless, we believe that the insights gained may reflect the perspectives of a broader population. Additionally, all interviews were in English; those who don’t speak English did not reach out to participate, meaning their experiences and viewpoints on the research issue remain unaddressed.

**Conclusion and recommendation for future research**

In conclusion, our study underscores the significant role played by cultural beliefs and perceptions, and healthcare professional’s attitudes in disclosing women’s feelings to healthcare professionals and in shaping help-seeking behaviours regarding perinatal mental health. The intricate interplay between individual knowledge, cultural norms, and societal expectations stresses the importance of screening and identification of common perinatal mental health disorders by healthcare professionals to access support and care as early as possible during pregnancy and before the deterioration of the symptoms and affecting the wider family. By addressing these barriers, healthcare systems can ensure equitable access to perinatal mental healthcare and enhance outcomes for women from diverse ethnic backgrounds. Future research should continue to explore the multifaceted aspects of cultural beliefs and perceptions, further informing evidence-based interventions and support systems for women experiencing perinatal mental health issues.

**List of Abbreviations**

GP: General Practitioner

NIHR: National Institute of Health Research

NHS: National Health Services

PAG: Participant Advisory Group

PMH: Perinatal Mental Health

VCS: Voluntary and Community Service

**References**

1. Meltzer-Brody S, Stuebe A. The long-term psychiatric and medical prognosis of perinatal mental illness. Best Pract Res Clin Obstet Gynaecol. 2014;28(1).

2. Womersley K, Ripullone K, Hirst JE. Tackling inequality in maternal health: Beyond the postpartum. Futur Healthc J. 2021;8(1).

3. Langham J, Gurol-Urganci I, Muller P, Webster K, Tassie E, Heslin M, et al. Obstetric and neonatal outcomes in pregnant women with and without a history of specialist mental health care: a national population-based cohort study using linked routinely collected data in England. The lancet Psychiatry [Internet]. 2023 [cited 2023 Aug 30]; Available from: https://pubmed.ncbi.nlm.nih.gov/37591294/

4. Prady SL, Pickett KE, Gilbody S, Petherick ES, Mason D, Sheldon TA, et al. Variation and ethnic inequalities in treatment of common mental disorders before, during and after pregnancy: Combined analysis of routine and research data in the Born in Bradford cohort. BMC Psychiatry. 2016;16(1).

5. Watson H, Harrop D, Walton E, Young A, Soltani H. A systematic review of ethnic minority women’s experiences of perinatal mental health conditions and services in Europe. PLoS One. 2019;14(1).

6. Flynn AB, Fothergill KE, Wilcox HC, Coleclough E, Horwitz R, Ruble A, et al. Primary Care Interventions to Prevent or Treat Traumatic Stress in Childhood: A Systematic Review. Vol. 15, Academic Pediatrics. 2015.

7. Pettman D, O’Mahen H, Blomberg O, Svanberg AS, von Essen L, Woodford J. Effectiveness of cognitive behavioural therapy-based interventions for maternal perinatal depression: a systematic review and meta-analysis. BMC Psychiatry. 2023;23(1).

8. Sockol LE, Epperson CN, Barber JP. A meta-analysis of treatments for perinatal depression. Vol. 31, Clinical Psychology Review. 2011.

9. Jankovic J, Parsons J, Jovanović N, Berrisford G, Copello A, Fazil Q, et al. Differences in access and utilisation of mental health services in the perinatal period for women from ethnic minorities - A population-based study. BMC Med. 2020;18(1).

10. Pilav S, De Backer K, Easter A, Silverio SA, Sundaresh S, Roberts S, et al. A qualitative study of minority ethnic women’s experiences of access to and engagement with perinatal mental health care. BMC Pregnancy Childbirth. 2022;22(1).

11. Anderson FM, Hatch SL, Comacchio C, Howard LM. Prevalence and risk of mental disorders in the perinatal period among migrant women: a systematic review and meta-analysis. Arch Womens Ment Health. 2017;20(3).

12. MacLellan J, Collins S, Myatt M, Pope C, Knighton W, Rai T. Black, Asian and minority ethnic women’s experiences of maternity services in the UK: A qualitative evidence synthesis. J Adv Nurs. 2022;78(7):2175–90.

13. Smith MS, Lawrence V, Sadler E, Easter A. Barriers to accessing mental health services for women with perinatal mental illness: Systematic review and meta-synthesis of qualitative studies in the UK. Vol. 9, BMJ Open. 2019.

14. NHS England. The NHS Long Term Plan [Internet]. Vol. 1.2, NHS England. 2019. Available from: https://www.longtermplan.nhs.uk/wp-content/uploads/2019/08/nhs-long-term-plan-version-1.2.pdf

15. Braun V, Clarke V. Using thematic analysis in psychology. Qual Res Psychol. 2006;3(2).

16. Boddy R, Gordon C, MacCallum F, McGuinness M. Men’s experiences of having a partner who requires Mother and Baby Unit admission for first episode postpartum psychosis. J Adv Nurs. 2017;73(2).

17. Conneely M, Packer KC, Bicknell S, Janković J, Sihre HK, McCabe R, et al. Exploring Black and South Asian women’s experiences of help-seeking and engagement in perinatal mental health services in the UK. Front Psychiatry. 2023;14.

18. Millett L, Taylor BL, Howard LM, Bick D, Stanley N, Johnson S. Experiences of improving access to psychological therapy services for perinatal mental health difficulties: A qualitative study of women’s and therapists’ views. Behav Cogn Psychother. 2018;46(4).

19. Edge D. Falling through the net - Black and minority ethnic women and perinatal mental healthcare: health professionals’ views. Gen Hosp Psychiatry. 2010;32(1).

20. Edge D. “It’s leaflet, leaflet, leaflet then, ‘see you later’”: Black Caribbean women’s perceptions of perinatal mental health care. Br J Gen Pract. 2011;61(585).

21. Cooper C, Spiers N, Livingston G, Jenkins R, Meltzer H, Brugha T, et al. Ethnic inequalities in the use of health services for common mental disorders in England. Soc Psychiatry Psychiatr Epidemiol. 2013;48(5).

22. Barnett P, Mackay E, Matthews H, Gate R, Greenwood H, Ariyo K, et al. Ethnic variations in compulsory detention under the Mental Health Act: a systematic review and meta-analysis of international data. The Lancet Psychiatry. 2019;6(4).

23. Wittkowski A, Zumla A, Glendenning S, Fox JRE. The experience of postnatal depression in South Asian mothers living in Great Britain: A qualitative study. J Reprod Infant Psychol. 2011;29(5).

24. Phillips L. Assessing the knowledge of perinatal mental illness among student midwives. Nurse Educ Pract. 2015;15(6).

25. Jomeen J, Glover L, Jones C, Garg D, Marshall C. Assessing women’s perinatal psychological health: Exploring the experiences of health visitors. J Reprod Infant Psychol. 2013;31(5).

26. Jones I, Chandra PS, Dazzan P, Howard LM. Bipolar disorder, affective psychosis, and schizophrenia in pregnancy and the post-partum period. Vol. 384, The Lancet. 2014.

**Declarations**

**Authors’ contributions:** The study was conceived by EM, HOM, AD, GF, SMT, and GC. The topic guide for semi-structured interviews was composed equally by all authors. Semi-structured interviews were carried out by EM. Revisions to the topic guide were discussed amongst all the authors. Transcripts were analysed by EM and AD. All authors provided feedback and guidance. EM wrote the manuscript, with editing and revisions suggested by the remaining authors. All authors have approved this manuscript.

**Acknowledgements:** The study team would like to thank all the participants for their contribution to this research. In addition we would like to thank our PAG for their support and advice during the course of the study.

**Funding**: This study/project is funded by the National Institute for Health Research (NIHR) Health Services and Delivery Research Programme (Ref: 17/49/38). The views expressed are those of the author(s) and not necessarily those of the NIHR or the Department of Health and Social Care.

**Availability of data and materials**: The datasets generated and/or analysed during the current study are not publicly available for confidentiality reasons but are available from the corresponding author on reasonable request.

**Ethics approval and consent to participate**: The study was approved by the Southwest - Central Bristol Research Ethics Committee (Reference: 19/SW/0218). All participants gave informed written consent for participation in the study and for publication of the project findings and written quotations prior to participation.

**Consent for publication:** Not Applicable.

**Competing interests**: The authors declare no competing interests.
